# Predictive value of quick surgical airway assessment for trauma (qSAT) score for identifying trauma patients requiring surgical airway in emergency room

**DOI:** 10.1186/s12873-018-0203-4

**Published:** 2018-11-29

**Authors:** Kei Hayashida, Shokei Matsumoto, Mitsuhide Kitano, Junichi Sasaki

**Affiliations:** 1 Department of Emergency and Critical Care Medicine, School of Medicine, Keio University, 35 Shinanomachi, Shinjuku-ku, Tokyo, 160-8582 Japan; 2 Department of Trauma and Emergency Surgery, Saiseikai Yokohamashi Tobu Hospital, 3-6-1 Shimosueyoshi, Tsurumi-ku, Yokohama, 230-8765 Kanagawa Japan

**Keywords:** Trauma, Emergency airway, Surgical airway, Difficult airway, Prediction, Intubation, Emergency room

## Abstract

**Background:**

A surgical airway is usually unpredictable in trauma patients. The aim of this study was to develop a predictable scoring system to determine the need for a surgical airway by using a database from a large multicenter trauma registry.

**Methods:**

We obtained data from the nationwide trauma registry in Japan for adult blunt trauma patients who were intubated in the emergency department. Based on a multivariate logistic regression analysis in the development cohort, the Quick Surgical Airway Assessment for Trauma (qSAT) score was defined to predict the need for a surgical airway. The association of the qSAT with surgical airway was validated in the validation cohort.

**Results:**

Between 2004 and 2014, 17,036 trauma patients were eligible. In the development phase (*n* = 8129), the qSAT score was defined as the sum of the three binary components, including male sex, presence of a facial injury, and presence of a cervical area injury, for a total score ranging from 0 to 3. In the validation cohort (*n* = 8907), the proportion of patients with a surgical airway markedly increased with increasing qSAT score (0 points, 0.5%; 1 point, 0.9%; 2 points, 3.5%; 3 points, 25.0%; *P* <  0.001). Multivariate analysis revealed that qSAT score was an independent predictor of surgical airway (adjusted OR, 3.19 per 1 point increase; 95% CI, 2.47–4.12; *P* <  0.0001). The qSAT score of ≥1 had a had a good sensitivity of 86.8% for predicting the requirement for surgical airway; while qSAT score of 3 had a good specificity of 99.9% in ruling out the need for surgical airway.

**Conclusions:**

The qSAT score could be assessed simply using only information present upon hospital arrival to identify patients who may need a surgical airway. The utilize of qSAT score in combination with repeated evaluations on physical finding could improve outcomes in trauma patients.

## Introduction

Trauma patients frequently require tracheal intubation during their initial resuscitation. Difficult tracheal intubation (DTI) is identified as a major cause of morbidity and mortality among trauma patients treated in the emergency department (ED) [1–6]. Surgical airway is an uncommon procedure in the ED but an important rescue method particularly in trauma patients with DTI when several attempts at orotracheal intubation (OTI) have failed [7]. Because poor airway management can result in catastrophic consequences, the assessments of both DTI and the need for a surgical airway before trying OTI have crucial roles in the management of trauma patients.

Initial airway management in trauma requires the emergent assessment of DTI and the necessity for a surgical airway as trauma patients often present with airway obstruction, respiratory failure, or shock on ED arrival. Both an early decision to intubate and rapid identification of the need to transition to the surgical airway from OTI are essential concepts in trauma care. However, although several scoring systems have been utilized to predict survival outcome and the need for massive transfusion in trauma patients [8–12], the prediction of DTI remains an imperfect science as the tests fail to predict some difficult intubations, and there is little research on scoring systems predicting the need for a surgical airway in trauma patients [13]. Therefore, we sought to develop a novel method to simply and quickly estimate the need for a surgical airway in trauma patients during the initial management.

The aim of this study was to examine the risk factors for surgical airway and to develop a new scoring system to predict the need for a surgical airway in trauma patients by using the database from a large, multicenter observational registry of trauma patients in Japan. We hypothesized that a novel scoring system, the Quick Surgical Airway Assessment in Trauma (qSAT) score, can be used to simply assess and identify patients who need a surgical airway based only upon information present upon hospital arrival.

## Materials and methods

### Study design and settings

The qSAT score presented here was retrospectively derived from the database of the Japan Trauma Data Bank (JTDB) using data retrieved between 2004 and 2014 in Japan. The details of the JTDB have been described elsewhere [14–16]. Briefly, the Japanese Association for Trauma Surgery (Trauma Registry Committee) and the Japanese Association for Acute Medicine (Committee for Clinical Care Evaluation) established the JTDB in 2003. The JTDB now includes 234 participating emergency hospitals from all over Japan, and most of them are approved as tertiary emergency centers by the Japanese government. The data was manually entered into a web-based data server using specific record sheets. The JTDB does not use the International Classification of Diseases (ICD). Diagnosis of injury is recorded according to the Abbreviated Injury Scale (AIS) using AIS 90 update 98. The original checklist items are used to register the surgical procedure codes of the JTDB. Surgical airway management was identified and defined as “cricothyroidotomy or tracheostomy” as reported in the emergency procedure section. This study was reported based on the recommendations of the STARD statement (“Standards for Reporting of Diagnostic Accuracy”) [17] for diagnostic accuracy studies.

### Patient selection

The inclusion criteria were 1) patients subjected to blunt trauma, and 2) patients who were intubated in the ED (either non-surgical or surgical tracheal intubation). The exclusion criteria were 1) age < 16 years, 2) patients having AIS 6 or 9 in any region, and 3) cardiopulmonary arrest upon hospital arrival.

### Data definition

The patterns of injury in body regions were simply divided into two categories: AIS ≤ 1 or AIS ≥ 2. Using the AIS score recorded in the database, we defined the appearance of trauma patients upon hospital arrival as follows: AIS ≤ 1 was defined as intact or minor injury, AIS ≥ 2 was defined as moderate-to-critical injury.

### Model development and validation

In the development phase, we reviewed data from blunt trauma patients recorded between January 2004 and December 2010. The following predefined potential predictors were evaluated: age, sex, Injury Severity Score (ISS), Revised Trauma Score (RTS), and whether patient had a moderate injury in each body region including head, face, neck, thorax, abdomen, spine, upper extremities, pelvis and lower extremities, and surface and cervical spine (C-spine). Then, multivariate logistic regression models were constructed to assess the associations of independent factors with surgical airway. The model was adjusted for age (16–59 years vs ≥ 60 years, with the patients divided based on the median age), sex, ISS, RTS, and whether patients had injury in each body region by using the forward elimination method. Forward elimination of variables from the model was set to a significance level of 0.10 and based on the probability of the likelihood-ratio statistic and maximum partial likelihood estimates. Then, the qSAT score was developed based on the results from the multivariate analysis.

In the validation phase, we reviewed data between January 2011 and December 2014. The accuracy of the qSAT score was described by sensitivity, specificity, relative ratio, positive likelihood ratio, and negative likelihood ratio. A multivariate logistic regression model was repeated to investigate the association of the qSAT score with surgical airway with adjustment for the same potential confounders used in the development phase.

### Sample size calculation

As we used a logistic regression model to construct a predictive score, the sample size had to be based on the events-per-variable ratio. This ratio had to be greater than 10. We had 110 and 106 events (patients with surgical airway) in the development and validation cohort, respectively. Therefore, we could construct a predictive model with 11 and 10 explanatory variables in the development and validation cohort, respectively [18].

### Statistical analysis

Categorical variables are presented as the number (frequency), and continuous variables are presented as the median (interquartile range [IQR]) because the duration of all continuous variables in our data showed non-normal distribution. The distribution of the continuous variables was compared using the Mann-Whitney U test. The chi-squared test or Fischer’s exact test was used for the comparison of binary variables. The linear trend across the levels of a variable was tested by the Cochran-Armitage trend test.

To improve the quality of the analyses, we performed multiple imputation to replace each missing value with a set of substituted plausible values by creating five filling-in copies to reduce bias caused by incomplete data, with the assumption that data were missing at random [19, 20]. Multivariate logistic regression models were constructed in each imputed copy, and the results of the five imputed copies were combined into one model, from which the statistical inference was taken [21, 22]. Goodness of fit for the logistic regression models was assessed using the Hosmer-Lemeshow test, and an adequate fit was assumed if *P* > 0.05. The odds ratio for the outcome was reported, along with *P*-values and Wald 95% confidence intervals (CIs). All *P* values are two-tailed, and *P* <  0.05 was considered significant. Statistical analyses were performed with IBM SPSS Statistics for Windows, Version 23.0 (IBM Corp., Armonk, NY).

## Results

### General characteristics

**Fig. 1:**
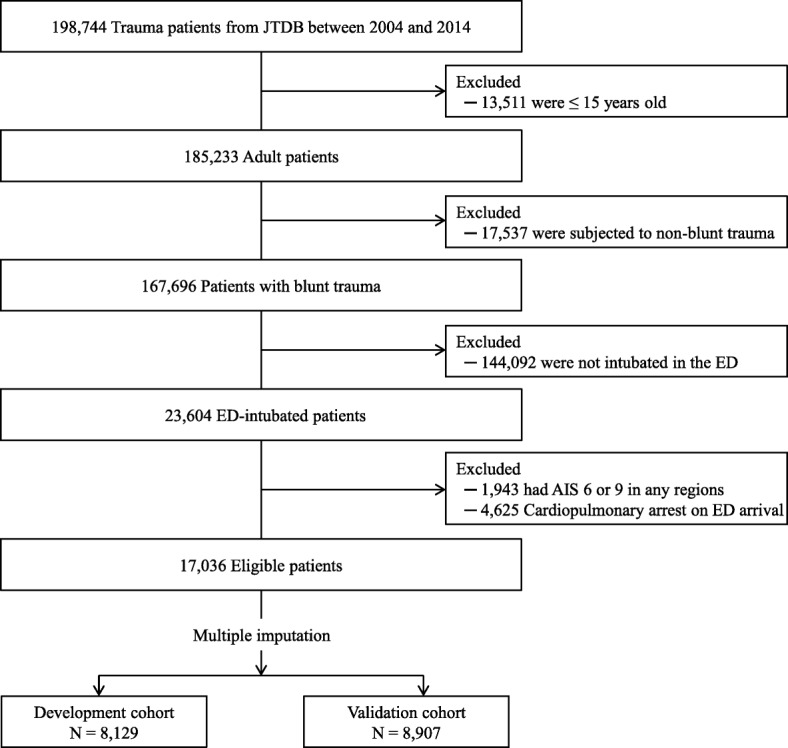
Patient selection. JTDB = Japanese Trauma Data Bank, ED = emergency department, AIS = Abbreviated Injury Scale

During the study period, 198,744 patients were identified. Of them, 17,036 trauma patients with intubation in the ED were eligible according to the inclusion and exclusion criteria (Fig. 1). Among the eligible patients, 8129 and 8907 patients were assigned to the development cohort and the validation cohort, respectively.

### Model development

Table 1 shows patient characteristics in the developmental and validation datasets. Of the 8129 patients in the development dataset, 8019 (98.6%) received OTI, whereas 110 (1.4%) received a surgical airway in the ED. Univariate analyses revealed that the following potential predictors were significantly associated with a surgical airway: younger age, higher proportion of males, lower proportion of having abdominal injury, and higher proportion of having injury in the following regions: face, neck, and C-spine.

**Table 1:** Patient Characteristics in Development Dataset (*N* = 8129) and Validation Dataset (*N* = 8907)

Variable	Development Dataset			Validation Dataset		
	Surgical Airway Management			Surgical Airway Management		
	No	Yes	*P*-value	No	Yes	*P*-value
N (%)	8019 (98.6)	110 (1.4)		8801 (98.8)	106 (1.2)	
Age, years, median (IQR)	57 (34–71)	44 (26–66)	0.001	61 (38–74)	50 (27–70)	0.006
Age group			0.026			0.056
16–59 y	4308 (53.7)	71 (64.5)		4245 (48.2)	61 (57.5)	
≥ 60 y	3711 (46.3)	39 (35.5)		4556 (51.8)	45 (42.5)	
Missing	0 (0)	0 (0)		0 (0)	0 (0)	
Male sex	5678 (70.8)	91 (82.7)	0.006	6156 (69.9)	92 (86.8)	0.006
Missing	0 (0)	0 (0)		0 (0)	0 (0)	
*Vital signs on hospital arrival, median (IQR)*						
Systolic blood pressure, mm Hg	122 (90–151)	128 (105–150)	0.307	125 (93–153)	129 (96–155)	0.493
Missing	0 (0)	0 (0)		0 (0)	0 (0)	
Heart rate, bpm	95 (78–116)	101 (80–113)	0.399	93 (77–113)	100 (80–120)	0.061
Missing	135 (1.7)	1 (0.9)		143 (1.6)	4 (3.8)	
GCS score	8 (4–13)	8 (4–13)	0.933	8 (4–13)	7 (4–14)	0.669
Missing	277 (0.6)	4 (0.6)		292 (3.3)	7 (6.6)	
Variable	Development Dataset			Validation Dataset		
	Surgical Airway Management			Surgical Airway Management		
	No	Yes	P-value	No	Yes	*P*-value
Revised Trauma Score (RTS), median (IQR)	6 (5–7)	6 (4–7)	0.775	6 (5–8)	6 (5–8)	0.480
Missing	893 (11.1)	16 (14.5)		969 (11.0)	19 (17.9)	
Injury Severity Score (ISS), median (IQR)	26 (20–36)	27 (20–34)	0.803	25 (19–35)	27 (18–35)	0.618
Missing	132 (16.4)	3 (2.7)		130 (1.5)	0 (0)	
Admission to the tertiary emergency hospitals	7056 (88.0)	92 (83.6)	0.164	7675 (87.2)	86 (81.1)	0.063
*Injured region, AIS score*						
Head, median (IQR)	4 (0–5)	3 (0–4)	0.002	1 (0–1)	1 (0–1)	0.507
AIS ≥ 2, n (%)	5417 (67.4)	64 (58.2)	0.037	5830 (66.2)	71 (67.0)	0.873
Face, median (IQR)	0 (0–0)	1 (0–2)	< 0.001	0 (0–0)	1 (0–2)	< 0.001
AIS ≥ 2, n (%)	1040 (13.0)	47 (42.7)	< 0.001	1237 (14.1)	44 (41.5)	< 0.001
Neck, median (IQR)	0 (0–0)	0 (0–0)	< 0.001	0 (0–0)	0 (0–0)	< 0.001
AIS ≥ 2, n (%)	58 (0.7)	11 (10.0)	< 0.001	64 (0.7)	8 (7.5)	< 0.001
Chest, median (IQR)	0 (0–4)	0 (0–4)	0.882	0 (0–1)	0 (0–1)	0.539
Variable	Development Dataset			Validation Dataset		
	Surgical Airway Management			Surgical Airway Management		
	No	Yes	*P* value	No	Yes	*P* value
AIS ≥ 2, n (%)	3750 (46.8)	50 (45.5)	0.785	4039 (45.9)	50 (47.2)	0.793
Abdomen, median (IQR)	0 (0–0)	0 (0–0)	0.012	0 (0–0)	0 (0–0)	0.245
AIS ≥ 2, n (%)	1853 (23.1)	14 (12.7)	0.010	1891 (21.5)	18 (17.0)	0.261
Spine, median (IQR)	0 (0–0)	0 (0–2)	0.002	0 (0–0)	0 (0–1)	0.261
AIS ≥ 2, n (%)	1438 (17.9)	32 (29.1)	0.003	2090 (23.7)	29 (27.4)	0.385
Upper extremities	0 (0–1)	0 (0–1)	0.097	0 (0–1)	0 (0–0)	0.231
AIS ≥ 2, n (%)	1623 (20.2)	27 (24.5)	0.265	1919 (21.8)	19 (17.9)	0.336
Pelvis and lower extremities, median (IQR)	0 (0–3)	0 (0–2)	0.104	0 (0–0)	0 (0–0)	0.518
AIS ≥ 2, n (%)	3043 (37.9)	33 (30.0)	0.088	3235 (36.8)	35 (33.0)	0.427
Surface, median (IQR)	0 (0–0)	0 (0–0)	0.558	0 (0–0)	0 (0–0)	0.260
AIS ≥ 2, n (%)	23 (0.3)	1 (0.9)	0.232	26 (0.3)	0 (0.0)	0.575
Cervical spine, median (IQR)	3 (2–4)	3 (2–4)	0.510	0 (0–0)	0 (0–4)	0.440
AIS ≥ 2, n (%)	652 (8.1)	24 (21.8)	< 0.001	920 (10.5)	24 (22.6)	< 0.001

Data are number (%) or median (IQR). *GCS* Glasgow Coma Scale, *AIS* Abbreviated Injury Scale

The results of the multivariate regression are presented in Table 2. They revealed that a surgical airway in the ED was significantly associated with male sex (adjusted OR, 1.68; 95% CI, 1.04–2.71; *P* = 0.045), having a facial injury (4.75; 3.19–7.09; *P* <  0.001), having a neck injury (10.83; 5.27–22.24; P <  0.001), and having a C-spine injury (2.58; 1.60–4.18; *P* <  0.001).

**Table 2:** Multivariate Logistic Regression Analysis for Surgical Airway in the Development Dataset

Variables	Adjusted OR	95% CI	*P* value
Neck injury (AIS of 2 or higher)			
Yes	10.83	5.28–22.23	< 0.001
No	Reference	–	–
Face injury (AIS of 2 or higher)			
Yes	4.83	3.24–7.21	< 0.001
No	Reference	–	–
Cervical spine injury (AIS of 2 or higher)			
Yes	2.63	1.62–4.26	< 0.001
No	Reference	–	–
Male sex	1.67	1.01–2.76	0.047
ISS (per 1 point increase)	0.99	0.97–1.00	0.104
RTS (per 1 point increase)	0.96	0.85–1.08	0.483
Age group			
16–59 y	0.82	0.54–1.23	0.34
≥ 60 y	Reference	–	–
Admission to the tertiary emergency hospitals	0.59	0.35–1.00	0.05

*OR* Odds ratio, *CI* Confidence interval, *AIS* Abbreviated Injury Scale, *ISS* Injury Severity Score, *RTS* Revised Trauma Score. The Hosmer-Lemeshow tests were used to assess the goodness of fit of the model (*P* > 0.05)

Of note, in the AIS coding, C-spine injury is categorized as a spinal injury (AIS Region 6) but not as a neck injury (AIS Region 3). As it is too difficult to clinically distinguish between a neck injury and C-spine injury during the primary assessment of the patient on arrival, we defined either neck or C-spine injury as a “cervical area injury”. Then, favoring simplicity over accuracy, each variable was converted into a simple binary score, irrespective of the regression coefficients according to a previous report [22]. Thus, the qSAT score was defined as the total of three component scores: male sex (female, 0 points; male, 1 point), presence of a moderate-to-critical facial injury (no, 0 points; yes, 1 point), and presence of a moderate-to-critical cervical area injury (no, 0 points; yes, 1 point) for a total score ranging from 0 to 3.

### Validation

**Fig. 2:**
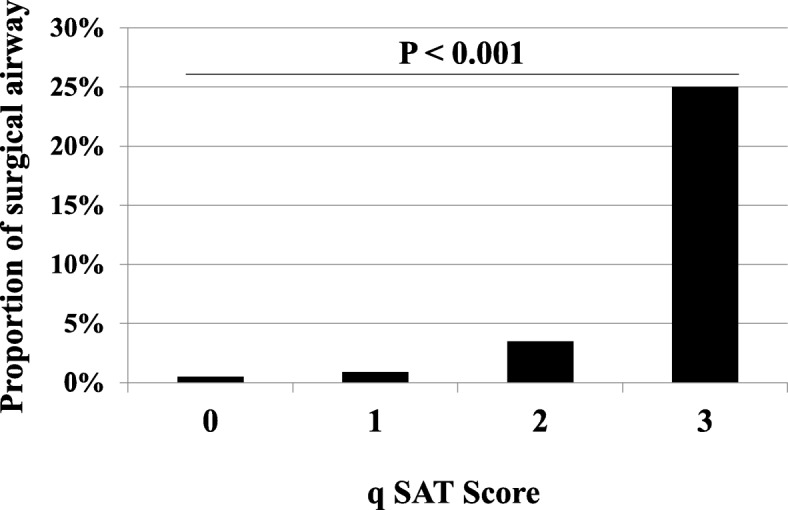
Association of qSAT scores with the probability for surgical airway management in the validation dataset

Of the 8907 patients in the validation cohort, 8801 (98.8%) received OTI, whereas 106 (1.2%) received a surgical airway in the ED (Table 1). Univariate analyses revealed that patients with a surgical airway were younger, a higher proportion were male, and had an AIS ≥ 1 in the face, neck, and C-spine regions compared to patients without a surgical airway. Figure 2 shows the association of qSAT scores with the probability for surgical airway in the validation dataset. The proportion of patients requiring a surgical airway markedly increased with increasing qSAT score (0 points, 0.5%; 1 point, 0.9%; 2 points, 3.5%; 3 points, 25.0%; *P* <  0.001, Fig. 2). The diagnostic accuracy for different ranges of the qSAT score in the validation cohort is indicated in Table 3. These findings suggest that the qSAT score of ≥1 had a good sensitivity of 86.8% for predicting the requirement for surgical airway; while qSAT score of 3 had a good specificity of 99.9% in ruling out the need for surgical airway.

**Table 3:** Diagnostic Performance of qSAT Score for Prediction of Surgical Airway in the Validation Cohort

qSAT	Sensitivity	Specificity	RR	LR+	LR-	*P* value
≥ 1	0.868 (0.791−0.920)	0.290 (0.289−0.291)	2.66 (1.53−4.63)	1.22 (1.11−1.30)	0.46 (0.28−0.72)	< 0.001
≥ 2	0.443 (0.353−0.537)	0.864 (0.863−0.865)	4.91 (3.37−7.15)	3.26 (2.58−3.99)	0.64 (0.54−0.75)	< 0.001
= 3	0.038 (0.015−0.074)	0.999 (0.998−0.999)	21.79 (8.73−44.34)	27.28 (9.48−79.89)	0.96 (0.93−0.99)	< 0.001

LR+ = likelihood ratio for positive results, LR- = likelihood ratio for negative results. Quick Surgical Airway Assessment for Trauma (qSAT) score was defined as the total of the three component scores, including male sex (female, 0 point; male, 1 point), presence of the facial injury (no, 0 point; yes, 1 point), and presence of the cervical area injury (no, 0 point; yes, 1 point), for a total score ranging from 0 to 3

In the multivariate analysis (Table 4), the qSAT score was an independent predictor of surgical airway (adjusted OR, 3.19 per 1 point increase; 95% CI, 2.47–4.12; *P* <  0.0001; Hosmer-Lemeshow test, *P* > 0.05).

**Table 4:** Multivariate Logistic Regression Analysis for Surgical Airway in the Validation Dataset

	Adjusted OR	95% CI	*P* value
qSAT score	3.19	2.47–4.12	< 0.0001
Admission to the tertiary emergency hospitals	0.56	0.34–0.93	0.026
RTS (per 1 point increase)	1.01	0.89–1.13	0.928
ISS (per 1 point increase)	0.99	0.98–1.01	0.834
Age group			
16–59 y	0.85	0.58–1.25	0.433
≥ 60 y	Reference	–	–
Male sex	1.54	1.15–2.07	0.141

Quick Surgical Airway Assessment for Trauma (qSAT) score was defined as the total of the three component scores, including male sex (female, 0 point; male, 1 point), presence of the facial injury (no, 0 point; yes, 1 point), and presence of the cervical area injury (no, 0 point; yes, 1 point), for a total score ranging from 0 to 3. *AIS* Abbreviated Injury Scale. RTS revised Trauma Score, *ISS* Injury Severity Score. The Hosmer-Lemeshow tests were used to assess the goodness of fit of the model (*P* > 0.05)

## Discussion

The need for a surgical airway in trauma patients is usually unpredictable, and a surgical airway procedure is often promptly required [23]. To the best of our knowledge, this is the first report of the development of a new scoring system, the qSAT score, to predict the need for a surgical airway in trauma patients using data from a large multicenter cohort study of trauma patients in Japan. A high qSAT score was significantly associated with an increased likelihood for a surgical airway with adjustment for potential predefined confounders. Our findings highlight the important possibility that the qSAT score may be useful to avoid the risk of major complications during emergency airway management in a population of trauma patients. Since the q-SAT score should not be used as the single definitive test for prediction of surgical airway, the scoring system that predicts the need for a surgical airway can improve outcomes in these patients.

Emergency airway care is indispensable in the management of trauma patients presenting to the ED. The incidence of failed intubation in the ED is 0.5–1.1%, which is not common but higher than the rate of 0.05–0.35% in the operating room [24, 25]. Poor airway management has been recognized as a serious concern for decades, emphasizing the need for careful airway assessment and thereby, the quick and accurate prediction for a surgical airway in the trauma bay. However, there have been many attempts to develop a method to predict a difficult airway even in the ED, but none has been found. Of note, despite its clinical importance, there have been very few studies conducted on a predictable scoring system for DTI and the need for a surgical airway in trauma patients because the incidence of attempting a surgical airway in the ED is quite low [24–26]. The present study addressed this knowledge gap by evaluating the impact of a simple airway assessment tool derived from a large cohort of trauma patients in Japan.

Maxillofacial and neck trauma may directly affect the airway resulting in compromise [27, 28]. In Japan, orotracheal intubation (i.e. direct laryngoscopy or airway adjuncts) with manual in-line stabilization is recommended as standard of the initial airway management for the patients with actual or potential cervical spinal cord injury, according to the Japan Advanced Trauma Evaluation and Care (JATEC) guideline. The surgical airway management should be considered in patients who have unsuccessful intubation for two times. The present study showed that cervical area injury and facial injury were significantly associated with a surgical airway. These predictors may be clinically plausible because these are accompanied by impairment in visualizing the vocal cords and epiglottis, which is very important to the success of OTI during laryngoscopy. Facial injuries commonly interrupt the visual field of the laryngeal pharynx due to edema, hemorrhage, or bony destruction. In fact, several studies on severe maxillofacial trauma reported that many patients (17–60%) required an emergent surgical airway [29–31]. Similarly, laryngotracheal injury, which includes cervical area injury, frequently (15–74%) requires an advanced airway [32, 33]. In addition, patients with cervical area injury require immobilization of the cervical spine, which also causes difficulties in airway management. Meanwhile, a surgical airway less affects the risks related to cervical spine instability.

A secondary important finding in this study is that we firstly showed significant associations between a surgical airway and the severities of injury in the face, neck, and C-spine (*P* <  0.001 for all). To our knowledge, the severity and combination of these injuries have not been shown previously to be closely associated with DTI. In the previous study showing the surgical airway rates of 0.7% among prehospital traumatic patients, severe burns and significant head and neck injuries were identified as candidates for surgical airway [34]. Another study has shown that with presence of anesthesiologists, the surgical airway rates were 0.3%; and head and neck injuries were identified as significant risk factors for surgical airway [35, 36]. These findings were consistent with our results. Further, consistent with previous reports [33, 37, 38], the overall cohort in this study showed that neck and C-spine injuries were found at a high rate in patients with facial injuries (37.4% [86/3932] and 29.4% [489/3932], respectively). Our findings suggest that considerable careful observation of the face and neck area should be undertaken by the primary treating physicians in particular during the initial management of blunt trauma.

In Japan, almost all of ED airway managements including the surgical airway procedure are performed by emergency physician, but some of them are performed by anesthesiologist, depending on local protocols. In particular, almost all of surgical airway technics are provided by emergency physicians in the secondary and tertiary emergency hospitals. The initial management for trauma patients is standardized based on the JATEC guideline that consists of a primary survey and a secondary survey, which has gained wide acceptance for trauma management in Japan. During the initial airway management in ER, the surgical airway should be considered when orotracheal intubation is failed two times according to the JATEC guideline. Meanwhile, many guidelines suggest that when a difficult airway is anticipated, an awake intubation should be performed [39, 40]. Nevertheless, most emergency physicians have limited experience with awake intubation technique [41], while emergency physicians have considerable experience with rapid sequence intubation (RSI) to achieve airway control in the ED. [42] However, as it requires immediate sedation and a neuromuscular blocking agent, RSI is potentially dangerous in trauma patients. Thus, when a patient is anticipated to have a high risk of airway trouble, it is likely that a surgical airway could be a good option without routinely and persistently attempting OTI.

The qSAT was developed to quickly predict the need for a surgical airway using information rapidly available upon hospital arrival. In fact, it is difficult to accurately evaluate AIS severities of injury during initial emergency care. However, because the information required by the qSAT can be easily assessed by the primary treating physician upon the patient’s arrival, our finding that the qSAT is an independent predictor of a surgical airway has important clinical benefit. Moreover, our study splitting the overall cohort by time periods and developing a model using data from one period and evaluating its performance with data from another period (temporal validation) is, statistically speaking, a stronger approach than a study randomly splitting a single data set into model development and model validation data sets [43]. These indicate that the qSAT may be a useful and robust scoring system to alert the physician to the need for a surgical airway in trauma patients.

Our analyses indicated that male sex was risk factors of surgical airway management in the ED. This may be due to sex being a confounder of parameters not observed, and this observation is in agreement with previous papers identified male sex was a risk of difficult tracheal intubation during the perioperative period [44, 45].

There are several limitations in the current study. First, some patients with DTI could receive an alternative OTI other than a surgical airway, although the alternatives such as retrograde, laryngeal tube airway, fiberoptic laryngoscopy, or bougies are not popular in the EDs in Japan. In fact, trauma centers in Japan are not common, and surgical expertise is not typically present in the ED throughout the day. In this study, we were not able to obtain the information regarding airway adjuncts because of the lack of registered variables. However, since more than 80% of the eligible patients were transported to the tertiary emergency hospitals in this cohort (Table 1), it is conceivable that these airway adjuncts should be available in most case of difficult airway. Second, the JTDB did not provide detailed data concerning airway management such as the timing of OTI, number of OTI attempts before the surgical airway, and physiognomic features reported to be associated with difficult laryngoscopy including the size of the tongue relative to the pharynx (i.e., the Mallampati score), limited neck mobility, and short thyromental distance [44, 46–48]. Third, intubation is a procedure that depends greatly on the quality of the operator, which is difficult to assess in clinical studies. Fourth, when the score is 3, it increases the specificity but considerably compromises the sensitivity, leading to many false negative results. Besides, since the prevalence of surgical airway in the ED is quite low, the clinician should be aware that non-invasive intubation (i.e. endotracheal intubation) should be tried firstly even if the score is 3. Fifth, the generalizability of our experience is unknown. Although the JTDB is a multicenter registry which is a strength because the results can be better extrapolated to the general population, this study might be a limitation because of differences in the in-hospital procedures or protocols for the implementation of a surgical airway among hospitals. Thus, the role of qSAT score could be limited to patients who were transported to the hospitals participating in this study. Sixth, the data is missing on surgical airway as a rescue technique after failed intubations. With the increasing use of video laryngoscopes in EDs, it may be possible to improve the primary intubating attempt. Finally, as with any observational study, the associations between the predictive factors involved in the qSAT score and outcome (required surgical airway) does not necessarily prove causality and might be confounded by unmeasured factors. Thus, a prospective study on the predictive value of the qSAT to predict the need for a surgical airway is required.

## Conclusions

The qSAT, which requires the use only of information present upon hospital arrival, was developed to simply and quickly estimate the risk for surgical airway in trauma patients. Our data suggested that a higher qSAT score was associated with an increased likelihood of the need for a surgical airway in trauma patients treated in the ED in Japan. However, similar to the bedside tests of predicting difficult airway and difficult laryngoscopy, the qSAT score should not be relied upon as a single measure in deciding the need for surgical airway. Other possible clinical markers, the available expertise and resources should be taken into account in planning for airway management in patients who are at an increased risk of requiring surgical airway. Further clinical studies are warranted to validate the qSAT for predicting the need for a surgical airway in the ED and improving the mortality of trauma patients.

## Abbreviations

AIS: Abbreviated Injury Scale
Cis: Confidence intervals
C-spine: Cervical spine
DTI: Difficult tracheal intubation
ED: Emergency department
ICD: International Classification of Diseases
IQR: Interquartile range
ISS: Injury Severity Score
JATEC: Japan Advanced Trauma Evaluation and Care
JTDB: Japan Trauma Data Bank
OTI: Orotracheal intubation
qSAT: Quick Surgical Airway Assessment in Trauma
RSI: Rapid sequence intubation
RTS: Revised Trauma Score
